# Mortality Statistics

**Published:** 1874-03

**Authors:** 


### *Chicago Mortality Report for January, 1874.*

CHICAGO, February 17th, 1874.

*Editors Chicago Medical Journal :*

SIRS—I send you a copy of the Mortality Report of the City of Chicago, for January, with comparisons of some of the principal diseases with the preceding month ; also the corresponding month of last year.

I desire to call your attention to the comparison of small-pox of last month, with the corresponding month of last year. The diminution in the mortality is wholly due to the thorough system of vaccination and revaccination employed by the Board of Health, although not put into active operation until about six weeks since. It not only shows the protective power of vaccine (animal virus was used), but also demonstrates fully that vaccination is cheaper than funerals.

Respectfully,

BEN C. MILLER, *San. Supt.*

MORTALITY IN MONTH OF JANUARY, 1874.

| | |
|---|---|
| Accident, burns | 2 |
| “ crushed | 3 |
| “ drowning | 1 |
| “ by fall | 3 |
| “ thrown from buggy | 1 |
| “ overdose of morphine | 2 |
| “ “ “ laudanum | 1 |
| “ “ “ narcotic | 1 |
| “ by railroad | 3 |
| “ by scalding | 1 |
| “ by suffocation | 1 |
| Abscess, psoas | 1 |
| Aneurism of aorta | 1 |
| Aphthæ | 1 |
| Apoplexy | 4 |
| Bowels, obstruction of | 1 |
| “ ulceration of | 1 |
| Bladder, “ “ | 1 |
| Brain, congestion of | 7 |
| “ softening of | 2 |
| “ inflammation of | 9 |
| Bronchitis | 20 |
| “ capillary | 14 |
| Cancer | 2 |
| “ of breast | 1 |
| “ of stomach | 3 |
| Child-birth | 1 |
| Cholera infantum | 1 |
| Consumption | 53 |
| Convulsions | 86 |
| “ puerperal | 2 |
| Croup | 16 |
| “ membranous | 1 |
| Colic | 1 |
| Cyanosis | 2 |
| Debility, general | 3 |
| Delirium tremens | 1 |
| Diarrhœa, chronic | 2 |
| Diphtheria | 5 |
| Dropsy, general | 3 |
| “ cardiac | 2 |
| “ peritoneal | 1 |
| Dysentery | 1 |
| Embolia | 1 |
| Enteritis | 9 |
| Exhaustion | 1 |
| Epilepsy | 3 |
| Erysipelas | 3 |
| Fever, puerperal | 7 |
| “ remittent | 2 |
| “ scarlet | 5 |
| “ malignant | 2 |
| “ typhoid | 13 |
| Gangrene | 3 |
| Gastro Enteritis | 1 |
| Heart, disease of | 4 |
| “ organic disease of | 2 |
| “ hypertrophy of | 2 |
| “ valve disease of | 1 |
| Hemiplegia | 1 |
| Hepatitis | 2 |
| Hernia, strangulated | 1 |
| Hydrocephalus | 4 |
| Inanition | 9 |
| Intemperance | 1 |
| Influenza | 2 |
| Jaundice | 1 |
| Kidneys, Bright's disease of | 3 |
| Laryngitis | 1 |
| Liver, cirrhosis of | 1 |
| “ inflammation of | 1 |
| Lungs, congestion of | 9 |
| “ paralysis of | 2 |
| “ hemorrhage of | 3 |
| Malformation | 1 |
| Metritis | 2 |
| Measles | 1 |
| Meningitis | 14 |
| “ cerebro spinal | 9 |
| “ tubercular | 2 |
| Metro peritonitis | 1 |
| Œdema pulmonum | 2 |
| Old age | 15 |
| Ovariotomy | 1 |
| Paralysis | 2 |
| Perrotitis | 1 |
| Pelvic bone, enlargement of | 1 |
| Pericarditis | 1 |
| Peritonitis | 6 |
| “ puerperal | 1 |
| Pharyngitis | 1 |
| Pneumonia | 60 |
| “ typhoid | 2 |
| Pleurisy | 1 |
| Rheumatism | 1 |
| Small-Pox | 24 |
| Spina bifida | 1 |
| Suicide, drowning | 1 |
| “ laudanum | 4 |
| “ poison | 1 |
| “ shooting | 1 |
| Syncope | 1 |
| Tabes Mesenterica | 12 |
| Teething | 6 |
| Tumor on back | 1 |
| Ulceration of rectum | 1 |
| Uterus, hemorrhage of | 1 |
| Vitality deficient | 1 |
| Whooping cough | 5 |
| Total | 553 |

Premature births, 7 ; Still births, 70. Total 77

COMPARISON.

| | |
|---|---|
| Deaths in month of January, 1874 | 553 |
| " " preceding month in 1873 | 565 |
| Decrease | 12 |
| Deaths in corresponding month in 1873 | 711 |
| Decrease | 158 |

AGES.

| | | | |
|---|---|---|---|
| Under one year | 209 | Forty years to fifty | 38 |
| One year to two | 45 | Fifty " " sixty | 26 |
| Two years to three | 37 | Sixty " " seventy | 23 |
| Three " " four | 9 | Seventy " " eighty | 15 |
| Four " " five | 7 | Eighty " " ninety | 4 |
| Five " " ten | 19 | Ninety " " one hundred | 1 |
| Ten " " twenty | 12 | | |
| Twenty " " thirty | 53 | Total | 553 |
| Thirty " " forty | 55 | | |
| Colored | 5 | Males | 321 |
| White | 548 | Females | 232 |
| Total | 553 | Total | 553 |

Married, 162 ; Single, 391. Total 553

NATIVITIES, JANUARY, 1874.

| | | | |
|---|---|---|---|
| Austria | 1 | Holland | 3 |
| Belgium | 2 | Ireland | 52 |
| Bohemia | 4 | Italy | 1 |
| Canada | 8 | Norway | 11 |
| Native—Chicago | 70 | Scotland | 2 |
| Foreign, " | 224 | Sweden | 6 |
| United States, other parts | 72 | Switzerland | 4 |
| England | 10 | Unknown | 12 |
| France | 2 | | |
| Germany | 69 | Total | 553 |

Deaths daily, 18. Mean temperature, 29°. Rain fall, 4.47 inches.

MORTALITY BY WARDS, ETC., JANUARY, 1874.

| Wards. | No. Deaths. | Pop. in 1872. | Percentage. |
|---|---|---|---|
| 1 | 1 | 398 | one death in ---- |
| 2 | 4 | 2,174 | " " " ---- |
| 3 | 15 | 19,157 | " " " 1,277 |
| 4 | 12 | 16,832 | " " " 1,403 |
| 5 | 19 | 18,564 | " " " 977 |
| 6 | 51 | 31,371 | " " " 627 |
| 7 | 41 | 27,644 | " " " 674 |
| 8 | 30 | 30,253 | " " " 1,008 |
| 9 | 38 | 30,032 | " " " 790 |
| 10 | 16 | 16,624 | " " " 1,039 |
| 11 | 18 | 18,340 | " " " 1,019 |
| 12 | 16 | 20 876 | " " " 1,304 |
| 13 | 19 | 14,636 | " " " 770 |
| 14 | 16 | 15,892 | " " " 931 |
| 15 | 87 | 40,047 | " " " 460 |
| 16 | 34 | 19,099 | " " " 561 |
| 17 | 35 | 17,513 | " " " 500 |
| 18 | 29 | 19,977 | " " " 689 |
| 19 | 2 | 2,944 | " " " ---- |
| 20 | 11 | 5,023 | " " " ---- |
| | 494 | | |

Ratio of deaths to population in 1872, one death in 664.

| | | | |
|---|---|---|---|
| No. deaths brought forward | 494 | St. Luke's Hospital | 2 |
| Accidents | 19 | Protestant Orphan Asylum | 2 |
| Bridewell | 1 | Small-Pox Hospital | 4 |
| County Hospital | 10 | St. Joseph's " | 1 |
| Foundlings' Home | 3 | Suicides | 7 |
| Half Orphan Asylum | 1 | Mercy Hospital | 5 |
| Home for Friendless | 2 | | |
| Hospital, Alexian Bros. | 2 | Total | 553 |

CASES OF SMALL-POX REPORTED DURING JANUARY, 1874.

| Ward. | No. Cases. | Ward. | No. Cases. | Ward. | No. Cases. |
|---|---|---|---|---|---|
| 1 | 1 | 8 | -- | 15 | 18 |
| 2 | 1 | 9 | 1 | 16 | 8 |
| 3 | 3 | 10 | 2 | 17 | 7 |
| 4 | 1 | 11 | 2 | 18 | 8 |
| 5 | 1 | 12 | -- | 19 | 1 |
| 6 | 12 | 13 | 2 | 20 | -- |
| 7 | 9 | 14 | -- | Recently arrived, | 2 |
| Total | | | | | 79 |

| | |
|---|---|
| Cases during January, 1874 | 79 |
| " " December, 1873 | 122 |
| Decrease | 43 |
| Cases during January, 1873 | 270 |
| Decrease | 191 |

Compared with the preceding month there were—

3 less deaths by Accidents.
9 " " " Apoplexy.
2 " " " Cancer.
5 " " " Consumption.
6 " " " Debility, general.
3 " " " Diarrhœa.
16 " " " Enteritis.
3 " " " Epilepsy.
2 " " " Fever, remittent.
4 " " " " typhoid.
6 " " " Heart disease.
2 " " " Hydrocephalus.
3 " " " Inanition.
2 less deaths by Paralysis.
3 " " " Rheumatism.
12 " " " Small-Pox.
4 " " " Tabes mesenterica.
5 more " " Brain disease.
9 " " " Bronchitis.
11 " " " Convulsions.
8 " " " Croup.
4 " " " Lung disease.
8 " " " Meningitis.
2 " " " Old age.
22 " " " Pneumonia.
3 " " " Suicide.

Compared with the corresponding month of last year, there were—

15 more deaths by Accidents.
5 " " " Bronchitis.
3 " " " Consumption.
8 " " " Meningitis.
1 " " " Œdema pulmonum
1 " " " Paralysis.
1 " " " Teething.
3 less " " Apoplexy.
8 " " " Consumption.
2 " " " Croup.
3 less deaths by Delirium tremens.
9 " " " Diphtheria.
2 " " " Dropsy.
7 " " " Enteritis.
10 " " " Erysipelas.
13 " " " Pericarditis.
5 " " " Pneumonia.
2 " " " Pleurisy.
40 " " " Small-Pox.
1 " " " Tabes mesenterica.

